# Diagnosis of sarcoidosis in a patient with differentiated thyroid cancer and sudden rise of anti-thyroglobulin antibodies during follow-up

**DOI:** 10.1007/s12020-021-02613-4

**Published:** 2021-02-18

**Authors:** Feudo Vanessa, Inzani Frediano, Zagaria Luca

**Affiliations:** 1 Institute of Nuclear Medicine, Università Cattolica del Sacro Cuore, Rome, Italy; 2 Gynecopathology Unit, Fondazione Policlinico Universitario A. Gemelli IRCCS, Rome, Italy; 3 Nuclear Medicine Unit, Fondazione Policlinico Universitario A. Gemelli IRCCS, Rome, Italy

**Fig. 1:**
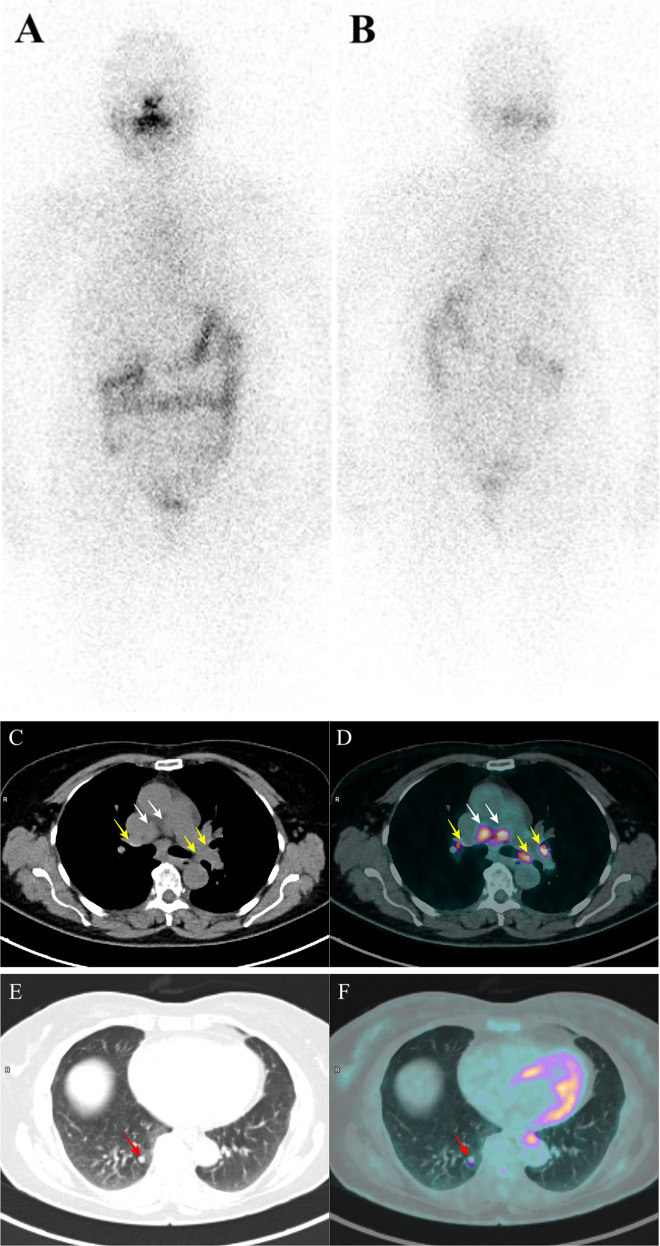

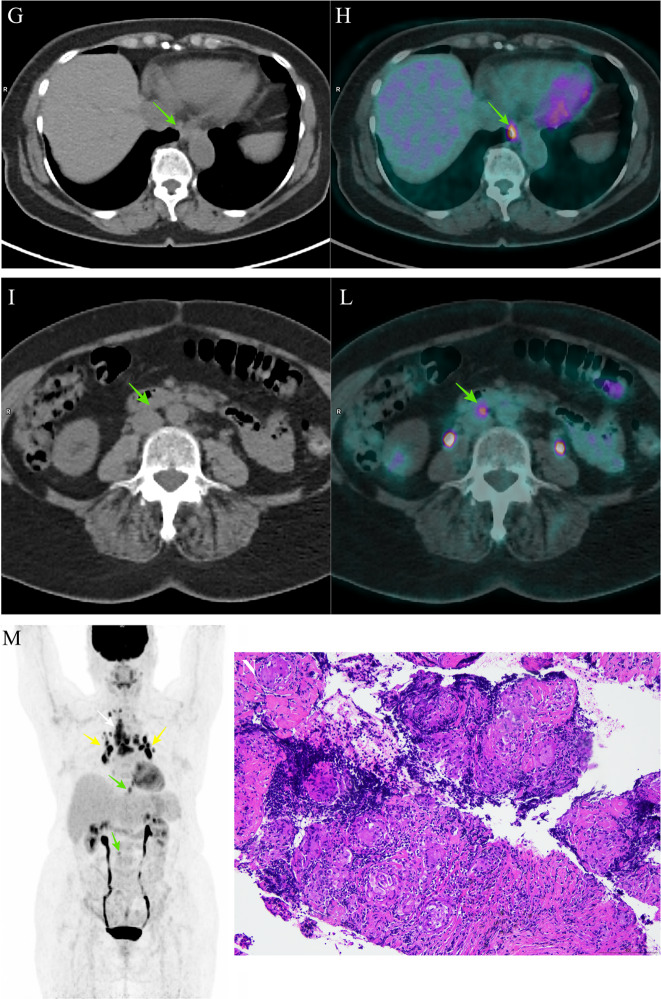
Diagnostic whole body scan with ^131^I revealed neither thyroid remnants nor ^131^I avid metastatic foci (a: anterior; b: posterior). ^18^F-FDG PET/CT (co-registered low dose CT (**c**, **e**, **g**, **i**), fused PET/CT images (**d**, **f**, **h**, **l**) and MIP (**m**)) showed bilateral hyper-metabolic mediastinal (white arrows) and hilar (yellow arrows) lymphadenopathies, pulmonary nodules (red arrows) and abdominal (green arrows) lymph nodes; histological examination of lymph node biopsy (**n**) showed non-caseating epithelioid granulomatous inflammation with multinucleated giant cells (Hematoxylin and Eosin)

A 70-years-old woman with a previous history of papillary thyroid cancer and loco-regional lymph nodes metastases came at our observation for a sudden rise of anti-thyroglobulin antibody (anti-Tg Ab) levels 14 years after initial treatment. She had been submitted to total thyroidectomy with right latero-cervical lymphadenectomy and successful thyroid ablation (2.3 GBq of ^131^I) in 2005. During subsequent follow-up, Tg levels were persistently undetectable and anti-Tg Ab levels were detectable but in the normal range (<60 UI/mL). On April 2019, anti-Tg Ab levels were >500 UI/mL, with undetectable serum Tg levels on levo-thyroxine (L-T4) suppressive therapy. Anti-Tg Ab levels, measured twice during the following 2 months, were persistently elevated. Because the presence of anti-Tg Abs can falsely reduce Tg levels and numerous studies have shown an increased risk of cancer recurrence associated with a new appearance or a rise of anti-Tg Abs, diagnostic examinations were performed. Neck ultrasound revealed no signs of relapse. A subsequent diagnostic whole body scan with 185 MBq (5 mCi) of ^131^I after L-T4 withdrawal revealed neither thyroid remnants nor ^131^I avid metastatic foci (Fig. 1a, b). With the suspicion of radioiodine non-avid lesions, ^18^F-fluorodeoxyglucose positron emission tomography/computed tomography (^18^F-FDG PET/CT) (Fig. 1c-m) was performed. It showed bilateral hyper-metabolic mediastinal (white arrows) and hilar (yellow arrows) lymphadenopathies, pulmonary nodules (red arrows) and abdominal (green arrows) lymph nodes. According to these findings, a differential diagnosis among cancer recurrence, lymphoproliferative disease, and multi-systemic inflammatory disease was posed. Patient was asymptomatic, she had not cough, dyspnea, chest pain, fatigue, weight loss, fever, itching, and muscle weakness; physical examination was normal. Whole blood count, gammaglobulines, alfa-globulines, beta-globulines, albumin, and lactic dehydrogenase were within the normal limit, whereas serum calcium levels were in the normal upper range.

For differential diagnosis an endoscopic ultrasound-guided fine needle biopsy of one intrathoracic lymph node was performed. Histological examination (Fig. 1n) showed non-caseating epithelioid granulomatous inflammation with multinucleated giant cells, consistent with sarcoidosis.

Sarcoidosis is an inflammatory disease characterized by an accumulation of inflammatory cells, called granulomas, into affected organs. Sarcoidosis can affect virtually any organ, but the most common localizations are the lungs, following by mediastinal and peripheral lymph nodes, skin, eyes, liver, spleen, nervous system, and heart [1]. The cause of sarcoidosis is unknown but it seems to be provoked by ﻿a combination of genetic influences and an exaggerated immune reaction to different triggers, as exogenous infective or non-infective antigens [1].

Different papers suggest the association between thyroid autoimmunity and sarcoidosis. Papadopoulos et al. [2]. evaluated the frequency and type of endocrine autoimmunity in 89 Swedish patients with sarcoidosis and found high frequency of thyroid autoimmunity in these patients compared to the general population. Ilias et al. [3]. evaluated thyroid function and the levels of anti-Tg Abs and antithyroid peroxidase autoantibodies (anti-TPO Abs) in patients with sarcoidosis compared to patients with chronic obstructive pulmonary disease; they found that in patients with sarcoidosis anti-Tg Abs were significantly increased, while anti-TPO Abs were within the normal range. Nakamura et al. [4]. compared anti-TPO Abs and anti-Tg Abs in patients with pulmonary sarcoidosis and in those without sarcoidosis and history of thyroid disease; they found high levels of these autoantibodies in patients with sarcoidosis, compared ﻿to controls. A study of Fallahi et al. [5]. demonstrated a high prevalence of other autoimmune disorders, such as sarcoidosis, in patients with chronic autoimmune thyroiditis, highlighting the possibility of a common pathogenesis. Other works showed a possible association/co-existence of papillary thyroid cancer and sarcoidosis. ﻿The pathophysiologic and clinical significance of anti-thyroglobulin autoantibodies in patients with sarcoidosis is not clear, but it may be interpreted as a non-specific feature of a systemic autoimmune activation, characteristic of sarcoidosis.

This case highlights that the increase of anti-Tg Abs in patients submitted to total thyroidectomy and subsequent treatment with ^131^I can be attributable to causes other than disease recurrence as sarcoidosis.
